# EEG Hyperscanning and Qualitative Analysis of Moments of Interest in Music Therapy for Stroke Rehabilitation—A Feasibility Study

**DOI:** 10.3390/brainsci12050565

**Published:** 2022-04-27

**Authors:** Gerhard Tucek, Clemens Maidhof, Julia Vogl, Astrid Heine, Matthias Zeppelzauer, Nikolaus Steinhoff, Jörg Fachner

**Affiliations:** 1 Josef Ressel Centre for Horizons of Personalised Music Therapy, Department of Health Sciences, Institute for Therapeutic Sciences, IMC University of Applied Sciences Krems, 3500 Krems, Austria; gerhard.tucek@fh-krems.ac.at (G.T.); clemens.maidhof@aru.ac.uk (C.M.); julia.vogl@fh-krems.ac.at (J.V.); astrid.heine@fh-krems.ac.at (A.H.); 2 Cambridge Institute for Music Therapy Research, Anglia Ruskin University, Cambridge CB1 2LZ, UK; 3 Department of Media & Digital Technologies, Institute of Creative Media Technologies, St. Poelten University of Applied Sciences, 3100 St. Poelten, Austria; matthias.zeppelzauer@fhstp.ac.at; 4 OptimaMed Neurological Rehabilitation Centre Kittsee GmbH, 2421 Kittsee, Austria; n.steinhoff@optimamed.at

**Keywords:** music therapy, stroke rehabilitation, moments of interest, process research, therapeutic relationship, mixed methods, EEG hyperscanning, social neuroscience, medical anthropology

## Abstract

Interdisciplinary research into the underlying neural processes of music therapy (MT) and subjective experiences of patients and therapists are largely lacking. The aim of the current study was to assess the feasibility of newly developed procedures (including electroencephalography/electrocardiography hyperscanning, synchronous audio–video monitoring, and qualitative interviews) to study the personal experiences and neuronal dynamics of moments of interest during MT with stroke survivors. The feasibility of our mobile setup and procedures as well as their clinical implementation in a rehabilitation centre and an acute hospital ward were tested with four phase C patients. Protocols and interviews were used for the documentation and analysis of the feasibility. Recruiting patients for MT sessions was feasible, although data collection on three consecutive weeks was not always possible due to organisational constraints, especially in the hospital with acute ward routines. Research procedures were successfully implemented, and according to interviews, none of the patients reported any burden, tiredness, or increased stress due to the research procedures, which lasted approx. 3 h (ranging from 135 min to 209 min) for each patient. Implementing the research procedures in a rehabilitation unit with stroke patients was feasible, and only small adaptations were made for further research.

## 1. Introduction

Music therapists focus on the interaction between client and therapist and how listening and creating music functions within the therapeutic relationship to achieve therapeutic goals.

The client is the focus of most case study research into the practice of music therapy; however, there is a lack of process-related data in the music neurosciences that inform us as to what is happening during music therapy and how responses to music are organised in situ [1]. There is now a well-documented body of evidence comprised of Cochrane reviews, systematic reviews, and scoping reviews on the effect of music therapy on outcome measures of different intervention arms of randomised control trials (RCTs), but neuroscience-informed process research into the mechanisms of change in music therapy is still underdeveloped [2]. Understanding how data in neuroscientific research is generated may help us to critically distinguish between models that explain the context of human behaviour and its ecological validity, i.e., how much it represents transferability into everyday life [3]. The laboratory is designed as an optimal measuring setting, reducing and controlling behaviour to elicit a target response, but it is here that the problem starts. If we want to record what happens in everyday life, then we need to have settings similar to everyday life [4]. The critique from some social scientists and anthropologists of lab-based behavioural-measuring procedures addresses the situation and process of measuring itself, which have an impact on the quality of the data [5]. Humanistic critiques have been based on the uniqueness and contextual nature of human experience, which is dependent on biographical time and place, as well as the uniqueness of the situation in which subjects are involved [6]. Being in a concert or listening to music on the radio adds the contextual dimension of personal experience in an ongoing situation onto perceptual processes. This influences intention and selection of what has been heard, selected, and perceived consciously during perception. The influence of the experimental setting in a laboratory is a critical issue, particularly when we consider how the subject regards his or her control of the situation.

“Not the objective control, as planned by the investigator, determines the changes of physiological measures but the subjectively experienced influence (control) on the process by the subject” [7].

The present research project aimed to identify and tread new pathways for personalised research in the therapeutic professions. To portray the clinical reality of the field, this project was designed to bring the laboratory into the clinical setting, rather than bringing patients into the laboratory, as described in our protocol paper [8]. Concrete music therapeutic processes are at the focus of this research. This should enable a deepened understanding of the mechanisms of relationship-oriented approaches to therapy based on music therapy, which greatly values the knowledge-based practice of relationship-oriented therapy models. 

“Simulated” conditions in music therapy research illustrate neither the reality nor the needs of therapists and patients and therefore hinder the transfer of research outcomes to clinical practice. One of the resulting consequences is the amplified time lapse between the creation of scientifically founded findings and their transfer into the clinical field [9]. Through the real-life application chosen for this study, research findings are more directly translated into the practical work and flow more quickly back into the field. 

Recent studies and reviews show the evidence and positive influence of music in neurorehabilitation after stroke or other acquired brain injuries [10,11,12]. After experiencing a stroke, the world is different than it was before. What worked before may now be limited, and must be learned and experienced anew. Music therapists grasp onto the individual capabilities, resources, and goals of their patients and integrate these into their therapeutic work. Here, we focus on relationship-oriented approaches, as an in-depth discussion of different approaches, their effectiveness, and evidence bases is beyond the scope of the current paper. The Krems Concept of Music Therapy [13] underpinning this study can be summarised as a relationship-oriented and resource-oriented biopsychosocial treatment concept. The personalised approach advocated through this concept does not place functional limitations after a stroke at the focus of the therapy, but rather sets the goal of physically, as well as psycho-emotionally, accompanying people after stroke through their recovery and new self-discovery process. The therapy can therefore include functional exercises and regulative elements, as well as mental processing of the event. 

In these relationship-oriented approaches, the therapist–patient relationship and joint musical participation are viewed as key factors of effectiveness [14,15,16]. This is similar to psychotherapy research, in which the importance of the therapeutic relationship and the therapist’s personality have been distinguished as the most significant factors [17,18,19]. The focus in relational approaches in health treatments lies on a jointly created interactive process. Within this process, of particular importance are those moments that have dialogical structures indicative of progress and change over the course of therapy [20,21,22,23]. These moments of interest (MOI) in the therapy can contain several aspects [24,25], such as a musical meeting or a shared awareness or perception of a (non)verbal interaction. The content of moments that are of importance and of interest for patients and therapists may vary according to the situation and person. Daniel Stern and the Boston Change Process Group [26] describe these moments in their research as “The Present Moment”. Several pieces of work have been dedicated to the exploration of qualitative descriptions of such meeting or present moments in music therapy [20,27,28]. The therapeutic relationship involves a highly dynamic interaction between patient and therapist [29]. When we interact with another person, our bodies and minds are not isolated, but rather embedded in a joint domain with the other person, in which we become a dyadic unit through mutual adjustments from moment to moment [30]. In clinical practice, music therapists experience that the impact of therapy manifests precisely in some of these individual moments. 

### Aims of the Feasibility Study

Interdisciplinary research into the underlying neural processes of music therapy (MT), specifically of moments of interest as well as the subjective experiences of patients and therapists, is largely lacking. One of the reasons for this is that there are no adequate adapted methods to investigate and accompany such fragile and highly context-sensitive situations such as music therapy sessions utilising improvisation and spontaneous therapeutic interventions [24]. Therefore, the aim of the current study was to assess the feasibility of newly developed procedures to study the personal experiences and neuronal dynamics of moments of interest during MT with stroke survivors in real-life situations.

Our overarching goal is to investigate which moments patients and therapists describe as interesting in the therapy and why, which interactive behaviours can be observed, and whether a “meeting” takes place in these moments. In the electroencephalogram (EEG), we are interested in which neurophysiological signatures can be identified, and whether there is a heightened synchronisation of brain and heart activity occurring in these moments between patient and therapist. We assume that despite the varying contents of MOIs, they may have underlying similarities in the dyadic neuro-cognitive-affective processes that are mirrored in the neurophysiological correlates, especially when these moments are consciously shared and even experienced as moments of meeting. It is expected this will be illustrated through the EEG in our research design and will be tested phenomenologically using case studies [23,31]. To facilitate this process, our interdisciplinary research team (made up of music therapists, anthropologists, and neuroscientists) developed a study protocol with a mobile lab, allowing for a real-life data collection setting directly in clinical practice with a higher case base [8]. The research should stay as ‘close to practice’ as possible [32] and adapt itself to the patients and the situation at hand [24], and not the other way around. Therefore, firstly, the MOIs must be identified during a therapy session. To achieve this, therapy patients and therapists will be interviewed, and the method of ‘participant observation’ applied to identify their individual subjective experiences, perspectives, and moments of interest. 

For practice-based research in real-life settings, it is crucial that the interaction between therapists and patients can take place naturally and be minimally influenced by any technology used for data collection. Moreover, the chosen imaging techniques must be able to record the temporal components of MOIs, which change in the range of milliseconds. These requirements of temporal resolution, though still enabling free movement, are currently only achieved with the EEG method. In our previous study [23], we were able to analyse segments that were pivotal for the therapeutic change in the session and described corresponding peaks and cross-correlations in the timecourse of a well-known neural marker of emotional processing (frontal alpha asymmetry, FAA). This study suggested that the FAA is well-suited to the study of interpersonal music-therapeutic processes and that it is sensitive to the temporal dynamics of dyadic emotional processes (for example, sharing emotional states, compassion, empathy). A further focus in the present study will be placed on the exploration of synchronised brain activity which, for example [33], could represent a neuronal mechanism allowing access to an internal state of another individual, and thus serve as the basis of shared emotions and a shared understanding [29]. 

A dyadic electrocardiogram (ECG) recording provides the additional possibility of investigating any synchronisation of cardiac parameters (heart rate, ECG amplitude) during MOIs. The analysis of dyadic music therapy processes as well as synchronisation and resonance phenomena via ECG allows for comparison to other investigations of therapy processes using markers of the autonomic nervous system. For example, in psychotherapy research, the non-verbal aspect of therapeutic interaction and social conditionality of situational therapy effects have been explored using socio-physiological investigations of heart rate covariations [34]. Currently, ECG synchronisation processes during psychotherapy are being investigated by windowed cross-correlations of ECG time series [35]. In music therapy, Neugebauer and Aldridge [22] had previously explored changes in ECG during dyadic improvisation. However, these investigations did not take place in clinical settings. Rather, the ECGs of healthy volunteers, including MT students and therapists, were measured in a clinical improvisation-like situation. Therapy segments, which were meaningful to the therapist according to their therapy summaries, were investigated using ECG and video for synchronous events. Detecting and analysing therapeutic relational processes using psycho-physiological data has been advanced in some fields (including neurorehabilitation) and shows the high potential of heart-rate variability (HRV) for demonstration and exploration of physiological processes in music therapy [36,37,38]. 

Prior to the current feasibility study, we conducted eight dyadic test measurements with healthy volunteers (music therapy students) in a controlled lab environment. The purpose of these recordings was to ensure sufficient synchronisation of the different data streams (audio, video, EEG, ECG), which is needed for microanalytically analysing non-verbal interactions such as blinking and mimicking. In addition, we needed to optimise the technical and practical procedures for use in a clinical context. After several adaptations, an approximate duration for data collection in the clinic could be estimated, and a feasibility study with four to six dyads (each with three music therapy sessions) was planned.

The goal of the feasibility study was to transfer and verify those experiences and procedures developed during the test measurements, including previously developed data collection and imaging methods for describing MOIs in the process of music therapy, and the technical procedures for EEG hyperscanning in the clinic. We did not know which moments the patients in phase C of their neurorehabilitation would find interesting, whether the planned interviews were practically feasible to carry out, and, similarly, whether the collected data could be included in the analysis, as was the case with the test measurements with healthy volunteers. As such, we had to test and, if necessary, adapt the data collection process and the analysis strategy with the clinical data. Salient to this process was getting to know the therapy rooms in which the measures would take place. The setup, camera positioning, and details such as lighting and background noise influence the evaluation and precision of the analysis. In particular, light and sound conditions can only be assessed in view of the video and audio materials. 

## 2. Materials and Methods 

### 2.1. Participants

In Austria, neurorehabilitation is structured into clearly defined phases (A to E), each linked with specific criteria, as defined by the Austrian Society for Neuro Rehabilitation (OeGNR, http://www.neuroreha.at/index.php/de/artikel-informationen/phasenmodell (accessed on 28 February 2022); for a comparison of stroke rehabilitation between European countries, see Putman and de Wit, 2009). According to this classification, patients in rehabilitation phase C are able to take part in therapies and activities for at least three hours per day. Therefore, we decided to set up our design for phase C patients. For our research questions, patients’ ability to verbally express themselves is crucial, whereas the time of the stroke was not important. 

Inclusion criteria for the study were (1) stroke as the main diagnosis, (2) rehabilitation phase C, (3) minimum age of 18 years, and (4) the ability to give consent. Exclusion criteria included aphasia or dysarthria, dementia (Mental State Test ≤ 24), post craniotomy status, clouding of consciousness, somnolence, acute infections, drug or substance abuse, hearing impairment, uncontrollable psychiatric issues (endangerment to self or others), or known allergies to the electrolyte gel or salt water.

The research team had access to patients’ medical data, such as the time of the stroke, affected brain region (using magnetic resonance imaging data), medication, and therapies. Information on the person’s handedness, age, and education was also collected. 

Ethical approval for this feasibility study, which was conducted in accordance with the Declaration of Helsinki, was given by the relevant ethics committee (Region Lower Austria GS1-EK-4/551-2018, Region Burgenland EK 88/2018). To participate, all participants went through a thorough explanation of the study with a member of the research team, after which they gave informed consent to take part. 

### 2.2. Data Collection

#### 2.2.1. Recruitment Sites

Data collection was carried out in two facilities in different states of Austria. The first was an inpatient neurological rehabilitation clinic (100 beds), where patients are regularly admitted and stay for three weeks for interdisciplinary rehabilitation. Acute treatment is not available in this facility. The second facility was a Department for Neurology in a university clinic (70 beds). In addition to acute medical treatment and diagnosis of various neurological illnesses, there are also inpatient spaces for phase C rehabilitation. These spaces are normally claimed by patients after the acute phase (A + B) or offered as a transition space until a rehabilitation space is available in the rehabilitation clinic. The duration of admission is therefore variable and not always predictable. 

**Figure 1:**
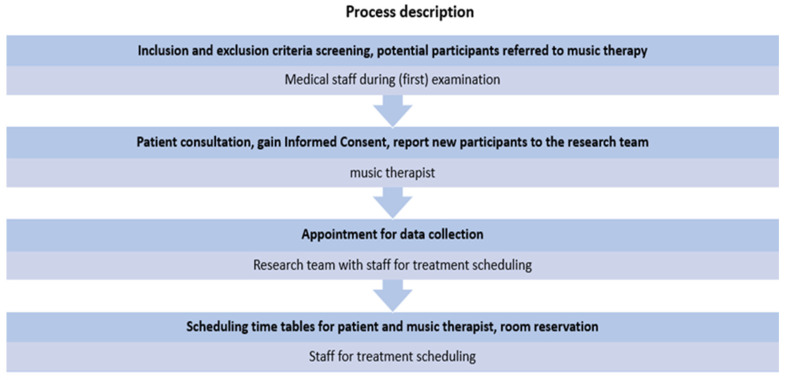
Recruitment process description.

Responsibility for individual steps in the recruitment process was shared between music therapists, medical doctors, and staff in the therapy department. At initial examinations or patient visits, doctors were asked to screen participants according to the inclusion and exclusion criteria and subsequently refer potential patients on to the music therapist. The music therapists carried out an extensive briefing about the research project and answered any questions the patients had. When informed consent was obtained, the music therapists informed the research team, who then scheduled an appointment for data collection (for an overview of recruitment processes, see Figure 1).

Three music therapy sessions per patient were recorded over the course of the therapy (1× per week). In addition to the music therapist, two Josef Ressel Centre (JRC) staff members were present during data collection. The tasks of staff members were to set up the technical equipment, prepare participants for the EEG/ECG, carry out the recordings, monitor patients, and carry out participant observation. 

#### 2.2.2. Feasibility of the Research Procedures

For this study, it was important to investigate the duration and acceptability of the investigation methods of the main study within the clinical context. The expected durations (based on our previous test measurements-see above) are outlined in our protocol article [8]. Each course of data collection was planned to last approximately 190 min and is illustrated in detail in Table 1. The actual results and average duration of the procedures are included here.

Inspired by Orsmond & Cohn [39], as part of the feasibility study, any issues and suggestions were recorded in a log sheet specifically created for this study (see Appendix A). Identified issues were also documented in field notes and addressed in detail in the interviews. The log sheet was filled out during and after every course of data collection and includes the total duration as well as the duration and acceptability of individual study procedures (for example, the EEG preparation, video rating, and MOI Interview), difficulties or issues in the planning, communication and implementation of data collection in the clinic, comprehensibility of our questions for participants, the observed burden for participants, as well as room for reflection and researchers’ comments. 

Additionally, an anthropologist carried out participant observations during the data collection and logged the course of events and any problem areas based on field notes. At the end of each course of data collection, participants were asked about their experiences of the research setting and its procedures and whether they would participate again, as well as whether they would recommend participation to others (see Appendix B). 

Throughout the study, feedback was collected from staff in the clinic (administrative personnel, doctors, carers in the unit, music therapists), and improvement suggestions for research processes as well as internal communication were taken on. In this way, the acceptability of the additional organisational tasks should be maintained, and the smooth running of the study ensured. 

#### 2.2.3. EEG and ECG Recording

For the hyperscanning of the patient–therapist dyad, mobile LiveAmp amplifiers (BrainProducts GmbH) were used. These send data wirelessly via bluetooth to two recording computers and also save it to the amplifiers’ internal memory cards. Simultaneously to the wireless EEG data transmission, a video is recorded by a camcorder, which is connected to the recording computer via an analogue–digital video converter. The recording software Brainvision Recorder (BrainProducts GmbH) receives both data streams and ensures a synchronous EEG-video recording.

In the first phase of the feasibility study, two different EEG caps were tested: 32-electrode gel caps (BrainProducts ActiCap) and 64-electrode saltwater-sponge caps (BrainProducts R-Net). The gel caps were placed on participants’ heads and subsequently filled with electrolyte gel to improve the conductance. For the use of a 64-electrode saltwater cap, two 32-channel LiveAmp amplifiers must be connected. The saltwater caps must soak in saltwater for 15 min before use and be put on the participants when saturation is achieved. As soon as a good impedance level was achieved, the dyad took their places for music therapy. EEG was recorded continuously throughout the therapy, with resting-state EEGs recorded at the beginning and end of the session (5 min each). As a control condition, and to investigate the role of visual information and synchronous music playing for inter-brain synchronisation, we asked participants after each session to play synchronously two drums with a tempo of 65 bpm (facing each other and facing away).

The EEG recordings were monitored by the researchers on laptops. Synchronously to the EEG data, the EEG amplifiers recorded two-channel ECGs. For this purpose, two electrodes were attached to participants’ upper body (right collarbone/left thorax) and synchronously recorded using the LiveAmp amplifier and the “Stimulus and Trigger Extension Box” (BrainProducts GmbH, Germany).

**Planned EEG and ECG Analyses**. The resting state EEGs will be analysed according to the power in various frequency bands and undergo a before–after comparison. This is used, among other reasons, for the neurometric assessment of the z-score deviations from a normative EEG database [40]. The analysis is based on the procedures implemented in the software “Neuroguide” (recognised by the American Food and Drug Administration), which has been proven effective for use in the clinical field [41,42]. The artefact cleaned, filtered and reliability checked (split-half, test-re-test) EEG will be split into two-second intervals, with each interval subjected to a Fast Fourier Transformation (FFT). The obtained power values of the individual frequency bands in each interval will be statistically tested for differences before and after music therapy using a *t*-test.

Emotionally relevant neural markers will also be investigated, including frontal alpha asymmetry (FAA) and frontal midline theta (FMT). The FAA describes the relative strength in the alpha frequency band (8–13 Hz) in the left and right frontal lobes and represents a neural marker of affective processes [43], which has previously been tested in the context of outcome-oriented music therapy [44]. In process-oriented music therapy research, our own research of receptive music therapy is the sole investigation in this area [23]. Here, we follow a similar approach, but with a higher case base and aim to extend FAA analyses with brain-to-brain coupling measures, such as the integrative coupling index, measuring directional phase coupling across time [45]. 

ECG measurements will be investigated using heartrate synchronisation and other cardiac parameters such as ECG amplitude. Due to the temporal synchronisation between ECG and videography, cardiac parameters can be considered during the analysis of MOIs.

#### 2.2.4. Participant Observation

An anthropologist conducted a participant observation. The advantage of this method lies in the proximity to the research participants, allowing context-specific phenomena to be captured directly. The manner and intensity of the researcher’s immersion in this process are dependent upon the observation style [46]. To maintain the natural feel of the setting while exerting minimal influence on the dyadic interaction between therapist and patient, an unstructured participant observation was chosen. The participant observer was introduced to patients as a member of the project staff who was responsible for monitoring recording technology and artefact control. Ongoing observations and (subjective) impressions were recorded in the form of field notes.

#### 2.2.5. Videography

We implemented a video setup in which a patient and therapist work together sitting across from each other. In addition to the synchronised EEG-video, three further video cameras were used. One of the cameras portrayed the “whole” of the therapy session (patient and therapist both visible), and an additional camera was aimed at the individuals, respectively (patient and therapist are each visible from the front).

The goal of this setup was, on one hand, to record the interaction between patient and therapist, and on the other hand, to record the facial expressions of the individual participants. Three GoPro Hero4 cameras were used; these recorded synchronously using an Arduino-based solution (https://mewpro.cc/en/ accessed on 28 February 2022). Both EEG amplifiers received a synchronisation marker that was manually elicited by the researcher through a button press. The button presses simultaneously activated a tone generator and an LED, which was recorded by the cameras. Thus, all data streams included a common synchronisation marker.

#### 2.2.6. Video Rating-MOI/MONI Selection

After the EEG measures and subsequent experience screening (see Table 2 below) the therapist and patient were separately asked to watch the video recording of the therapy session (on secured laptops) in its entirety, and to individually choose between three to five moments that were interesting for each participant. Although it can be assumed that more than three interesting moments may arise during therapy, out of practical reasons for the analysis, the number was limited to 3–5 MOIs. To implement a comparison condition, participants were also asked if they could indicate at least one Moment of No Interest (MONI). The exact start and end points of these moments were noted in a form. Participants could freely and independently watch the video, with the audio playing through headphones, and navigate throughout it. Staff assisted with the handling of the laptops as needed.

**Planned Analysis of the Video Data.** For the video analysis, the identified MOIs will be content-analysed in the video annotation software ELAN (Eudico Linguistik Annotator) [47]. In ELAN, video data from different cameras can be analysed simultaneously. Therefore, the different perspectives from the three cameras can be investigated both regarding the interaction between the participants (whole), as well as the individual events at the microlevel (facial expressions, eye contact, movement). The aim is to gain an overview and insight into the participant-chosen MOIs and to work out similarities and differences in the categories of observation. For the analysis, it is also of interest whether there are periods in which the MOI selection overlaps between therapist and patient. 

#### 2.2.7. Qualitative Interviews

The interviews (experience screening and MOI interview, see below in Table 2) were carried out separately with each participant, recorded with the help of a dictation device, and subsequently fully transcribed. Qualitative interviews allow a way into study participants’ cognitive and subjective world of experience. Methodologically, we oriented ourselves towards the episodic interview format, which can be attributed to Flick [48] and Helfferich [49]. Flick developed this interview method especially for the evaluation of subjective concepts, combined with aspects of the narrative interview [50], and supported by an interview guide. In this method, questions generated through narratives (stories) can be combined with open questions. Practically, this means that, although direct questions are posed, the interviewees are also continually prompted to describe events from their memories. This approach facilitated a sensitive interview adapted to patients’ and participants’ individual capabilities. Our chosen style of semi-structured and guide-supported interviews (see Appendix C) has already been implemented successfully in other studies with patients with stroke [51,52,53,54,55]. Three interviews were carried out at different times: the experience screening, the MOI Interview, and the feasibility interview. 

**Experience Screening**. In our personalised and participative approach, we are especially interested in the subjective angle, i.e., the views of therapists and patients and their lived experience of the therapy situation. Therefore, we carried out a short individual interview with each participant directly after the music therapy session (after the EEG measurements), in which they could freely recount how the session went for them and what they remembered. 

**MOI Interview.** After the independent selection of three to five MOIs, both participants were asked about their selections. The purpose of this was to examine the reasons for the selection, the meaning of the moments, and the qualitative experience thereof. After all, three moments were described, participants were asked if one of these moments could be identified as a moment of meeting (“… where a special connection between you and the person across from you occurred…”; see interview guide in Appendix C). If not, the participant was asked whether a moment of meeting had occurred at a different time point. When this was the case, patients gave a time indication and explanation for that moment. After the MOI discussion, both participants were given the opportunity to name at least one moment that was not interesting for them (Moment of No Interest-MONI). These later served as a comparison for the EEG analysis. Following each data collection, a feasibility interview was carried out; participants were asked how they had experienced the research setting, if the instructions were easily comprehensible, and whether the use of the computer was acceptable (see Table 2). After the last session, the patients were asked if they would take part in a study like this again in the future. 

**Planned Analysis of the Qualitative Interview Data.** Grounded theory [56,57] was chosen as the research approach for analysis of the interviews and participant observations. This theory-generating process is described as “grounded” because all interpretations arising from evaluation of the data are repeatedly considered in relation to the collected data, and thereby either confirmed or adjusted. Through this continuous grounding of interpretations, it is confirmed that a theory can be developed further in the research process and remains grounded in empiricism. The content of the transcribed interviews, as with the participant observations notes, will be investigated, coded, and categorised. Collected data will be analysed in three ways in the coding process, considering the field notes: open coding (aggregating phenomena into concepts and concepts into categories), axial coding (investigation of the categories for similarities and differences), and selective coding (isolating of a central category) [56].

This method allows for inductive development of a fitting theory or construct of the identified phenomena. The advantage of this method is the open and reflective approach.

#### 2.2.8. Capturing the Readiness for Therapy

Before each therapy session, participants filled out a questionnaire pertaining to their readiness for therapy [58]. This took about 2 min. Patients indicated how ready they currently felt for therapy (11 items), and therapists assessed how ready for therapy the patient seemed (6 items). The questionnaire used was developed in a parallel JRC project that aimed to identify preferred treatment times using focus groups and participative research methods for and with patients and therapists in Phase C neurorehabilitation. The questionnaire is currently being validated. Of interest for our project is whether there is a relationship between respective therapy readiness and the emergence and quality of MOIs.

#### 2.2.9. Combination of the Individual Methodical Approaches

A profile of the MOIs will be developed from the interview data, the participant observations, and the video analysis. Codes and categories from the interview analysis and participant observation will be used as qualitative information and attributed to specific MOIs and input into ELAN. This process allows for the analysis of subjective experience of the MOIs, with extracted descriptions contributing to a comprehensive profile of the MOIs. Subsequently, the temporal punctuation of the identified qualia will be analysed in conjunction with parallel running of the physiological data from EEGs and ECGs to describe neuronal and cardiac dynamics of the dyadic processes. This is how physiological markers of MOIs will be extracted and connected to the participants’ experience during music therapy [23].

## 3. Results

### 3.1. Recruitment and Sample Characteristics

Four study participants were recruited between October 2018 and June 2019 (mean post-stroke time of 11 months, range of 2 weeks to 29 months; mean age 62 years, range of 48 to 80). There was an interruption of three months due to a change of therapist. Four music therapists carried out recruitment talks at the chosen study sites. Two out of the six patients at both facilities who were asked refused to participate: one due to the presence of the researcher in the therapy room, and the other due to the videography. One patient agreed to participate on the condition that they would not lose time in other therapies because of their participation. 

Due to organisational issues such as holiday plans, early discharge or acute care referrals (see Table 2 and for Questions Q1–4 in Appendix B), only one patient was able to complete the three scheduled sessions. One patient was taken for acute medical investigation during the first data collection, meaning only the second session could be fully recorded; all others participated in two sessions. 

### 3.2. Access to Patients in the Respective Facilities

For the minimal disruption of regular operations at the facilities, data collection was carried out on one day per week. Due to the complexity of the mobile research setting and the duration of the research procedures (see Table 1), only one patient attended per data collection day. Data collection was ideally carried out in two to three consecutive weeks. As a result, not all potentially eligible participants were informed about the study and enrolled.

**Figure 2:**
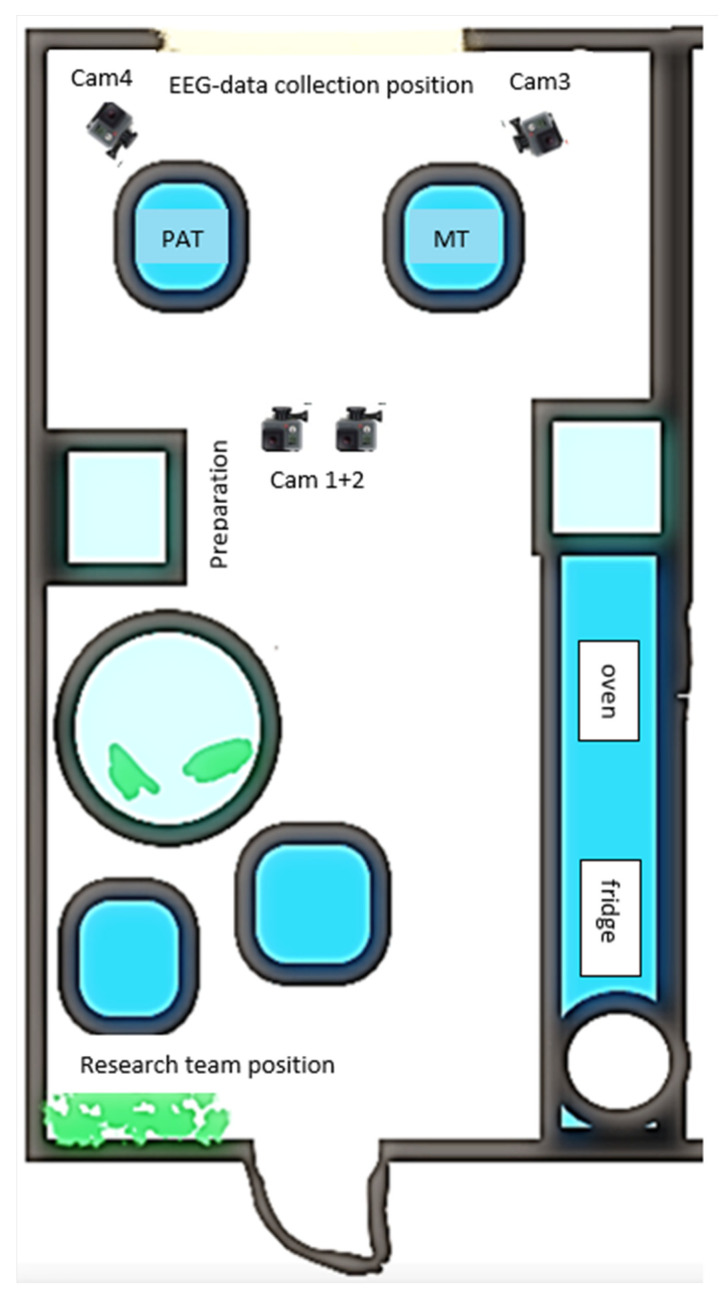
Room layout of the mobile lab in the rehabilitation clinic.

The music therapy room at the rehabilitation clinic turned out to be too small for the setup. Data recordings and therapy took place in a training kitchen that was reorganised into a therapy room (for room layout, see Figure 2). The therapy room in the acute ward was used by physio and music therapists and big enough for the setup. Equipment and furniture that were not used for the research were hidden behind a room divider.

### 3.3. Data Acquisition Methods

#### EEG and ECG

Initially, the equipment (EEG and video cameras) was set up in the study participants’ therapy room. This took an average of 43 min (range 30–60) for all four patients. Once set up was completed, patients were welcomed into the room. The application of the EEG caps, ECG electrodes, as well as synchronisation of the video and EEG technology took an average of 39 min (range 18–65). 

**EEG Caps.** The introduction of the new saltwater EEG caps noticeably shortened the preparation time by approximately 30 min. The application of the gel-based EEG caps (patients 1 and 2) took about 30–45 min to complete, whereas the saltwater caps (patients 3 and 4) were operational after about 5–15 min. Based on a first exploratory visual analysis of the EEG data quality, we could not detect any systematic differences. There were no detailed remarks in the interviews that the EEG caps were perceived as bothersome during music therapy sessions. The shorter application time for the saltwater caps was perceived by patients who had previously worked with the gel-based EEG caps as an alleviating factor. Any potentially uncomfortable wetness of the saltwater cap application was not noted. The experience of the saltwater cap was overall positive:

Patient: *“And this new cap was also better than the one that I wore at the beginning […].”*

Interviewer: *“So, the cap was more comfortable.”*

Patient: *“The cap was more comfortable than the old one. Even though I wore it for a long time, it didn’t bother me, only then at the end, it was maybe a little tight at the chin, but that was the only thing. Apart from that, not the cap itself. I didn’t feel it, as if I wasn’t wearing one at all.”*

**EEG Amplifier.** To be able to use the 64-electrode saltwater caps, two amplifiers must be connected for each person and worn by the participants (width × depth × height: 8.3 cm × 5.1 cm × 1.4 cm; 60 g per amplifier). Backpacks proved not to be practical for wearing the amplifier and the sensor and trigger extension box, as participants occasionally leaned on the backs of their chairs, placing increased pressure on the equipment and plugs. Equally problematic was the use of hip bags, as the heat dissipation was no longer adequately guaranteed. 

**Figure 3:**
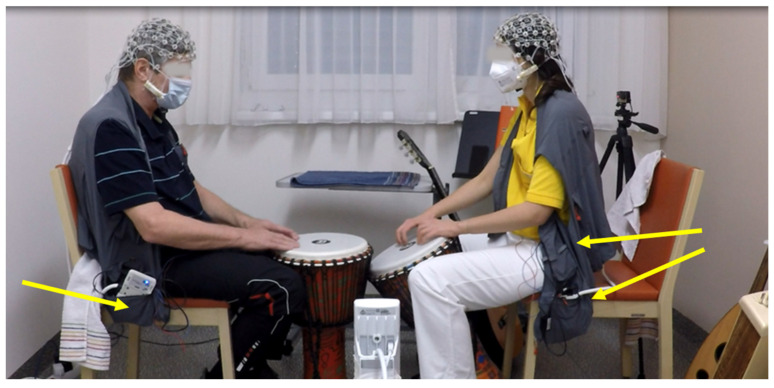
Patient and therapist wearing the Fisherman’s vests. The yellow arrows point to the respective pockets in which equipment was stowed.

To efficiently store all the technical appliances requiring close-to-body placement, and, above all, to make it comfortable for the participants, we used light and comfortable so-called Fisherman’s vests (see Figure 3), through which freedom of movement was not constrained and music therapy could proceed as usual. The equipment was placed into a pocket at hip-height. Two pockets were used for therapists: the amplifier was placed in the side hip-pocket, and the batteries, sensor, and trigger extension box were placed in the side chest-pocket. 

Further, of importance were concerns regarding the quality of the wireless data transmission. Other wireless devices (such as mobile phones, wireless speakers, etc.), as well as physical objects placed between the bluetooth receiver and the amplifiers, can interfere with the signals. Therefore, all electronic devices had to be switched off (as well as the oven) and physical objects in the transmission path were removed. To achieve a more stable bluetooth connection, we reduced the transmitted data by decreasing the EEG sample rate from 500 Hz to 250 Hz. However, lost data samples due to connection errors were common and need to be dealt with during the analysis stage.

### 3.4. Video

Synchronisation of the three GoPro cameras seemed not to work reliably or accurately with the Arduino-based solution (MewPro). A prerequisite for a simultaneous start of recordings of all GoPro cameras was a stable power supply voltage, which was not always supplied by the readily available USB-power adapters that we used. This resulted in unpredictable failures, e.g., of one camera to start recording. Using a dedicated USP (uninterruptible power supply) box (Eaton 3S 550 DIN) alleviated this problem. However, detailed analyses showed occasional asynchronies of the video and audio tracks, rendering the Arduino-based solution not suitable for our purposes. In addition, analyses of recordings made without the Arduino-based solution indicated considerable individual clock drifts in the GoPro cameras compared to the EEG. This was tested by comparing the intervals between two consecutive synchronisation impulses recorded in the EEG data streams (trigger signals) and video streams (tone/LED signals). The results of eight test recordings showed that, on average, the difference between intervals was around 140 ms, 140 ms, with a minimal difference of ca. 80 ms and a maximal difference of approx. 580 ms (corresponding to ca. 8 frames on average, min 4 frames, max 35 frames). Results showed no consistent or systematic speeding up or slowing down in the GoPro video data, which makes correcting the clock differences difficult. We therefore used the single-camera EEG-Video solution provided by the EEG manufacturer.

When interviewing the therapists, it turned out that, depending on the therapeutic intentions, the restriction of movement (based on the setup of the cameras mentioned above) was perceived as a potential influence on their therapeutic activities. We designed the video setup to properly capture a sitting patient and therapist and to enable a distinct recording of the upper limb and head to follow gaze and mimicking. 

However, being audio-video (AV) and EEG recorded was unfamiliar for the therapists at the outset, but later in the process, they did not notice the recording setup anymore. Interestingly, none of the patients mentioned the recording setup as problematic.

### 3.5. MOI Ratings

Patients selected between three and ten MOIs, but were always able to reduce these to the five to six most important ones (see Table 3 for a detailed overview of MOI/MONI selections and durations). However, the duration of MOIs varied significantly, ranging from 1 to 158 s. Finding MONIs proved to be difficult for patients, and no MONIs could be identified in half of the sessions. In addition, the duration of MONIs also varied considerably, ranging from 3 to 147 s (see Table 3). Importantly, the MOIs selected by the patients overlapped in seven out of eight sessions with therapist-selected MOIs, i.e., therapists and clients rated partly identical segments of the session as being interesting (durations of overlaps ranged from 1 to 35 s).

### 3.6. Interviews

All interviews were carried out as anticipated. The experience screening took place right after the therapy/recording session and lasted approximately 5 min for patients as well as therapists. The duration of the individual MOI interviews varied between 5 and 35 min for therapists (on average 18 min) and from 5 to 33 min (on average 18 min) for patients. In the MOI interviews, the participants seemed motivated and engaged. Preliminary analysis of the first dyad indicated that patients did not always reveal the personal meaning and emotional content of MOIs, meaning the interview stayed on a purely descriptive level. With further dyads, in response to purely descriptive accounts, the interviewer explicitly asked after personal meanings to also include this perspective.

In the feasibility interviews (see Table 2 for individual responses to questions 1 to 4; see also Appendix B for feasibility questions), patients showed interest and engagement and expressed their excitement for their participation in the research project. They fed back that they felt they were in good hands and looked after throughout the process. The instructions and questions were clear and comprehensible for the patients, despite any impairments. The patients experienced their participation in the research project as an enriching change to the everyday routines of the clinic. They described their willingness to participate in a similar study again if they felt they could offer a meaningful contribution to positive developments in neurorehabilitation after a stroke.

Feedback from one patient:

“*First of all, you think to yourself that maybe something will come of it that could somehow help other people. And of course, you also think of yourself, because you just think, maybe I will benefit from this myself through the music therapy that’s involved*.”

### 3.7. Patient Burden through the Research

None of the patient interviews indicated overload or fatigue because of the technology and applications. One therapist had the impression in a session that the patient was tired because of the lengthy application time (gel caps). From the perspective of the participating observer (JV), there were indications of patient fatigue and decreasing energy level when the application (especially of the gel caps) took a long time or when there were technical difficulties that needed to be solved urgently. 

Measurements from resting-state EEGs before and after music therapy sessions, the EEG recording during music therapy, and the comparison of back-and-forth synchronous drumming were not perceived as burdening or tiring. When the anticipated time frame ran over (for example, due to technical problems with setup prior to the therapy session), and participants had other appointments, or if the patient was noticeably tired or unfocused, the synchronous drumming was left out (see Table 2). 

## 4. Discussion

This feasibility study allowed us to test the procedures of a newly developed mobile setup with EEG, ECG, and video, as well as qualitative video ratings and interviews in a real clinical environment with persons after a stroke. 

An important observation was that patients did not experience any negative restrictions from the EEG application, nor increased burden due to the interview and video rating procedures, which was encouraging. Analysis of the feasibility interviews, intended to collect responses on the burden or stress of the participants, showed that patients were interested and curious about taking part in the study, and reported that the participation was a welcomed variety to their routines in rehabilitation and music therapy that was experienced as enriching their rehabilitation, as the following quote from a patient demonstrates:

*“Well, I think at the beginning I thought to myself, music therapy, how is that supposed to help me, because I’ve always been so focused on being able to move my arm and my leg and now somehow, I can see that music therapy can also contribute to improving my condition.”*

### 4.1. Selecting MOIs

To our knowledge, patients in neurorehabilitation have not previously participated in the analysis of their own sessions. Video analysis is a common tool used in music therapy practice and research, but the videos are mostly analysed by experts, and interviewing participants has not yet been included [59]. In microanalytic research, it is common to triangulate between different rater perspectives [60], and a recent scoping review suggested that the patient perspective is of importance for understanding change processes in therapy [2]. To realise this kind of research setting, a patient must consent to being videotaped (one screened patient did not agree to this; see 3.1 above), revisiting the therapy session, and they must be able to verbalise.

The interviews revealed that most patients were not used to reflecting and talking about their experiences after therapy. This was obvious in the first MOI interviews of patients, which were short superficial descriptions of the intervention, e.g., “This was when the therapist sang this song”—without talking about subjective experiences, thoughts, or emotions. After the first two sessions, the research team started to ask further questions to move more deeply into the qualitative experiences of each patient. The timing and duration of the interviews proved to be appropriate, only leading to minor changes in the interview guideline by posing the question of the experience of the research setting at the end.

The selection of MOIs was feasible for all participants. Patients did not report any stress during watching and rating the video, perhaps also due to the cooperative atmosphere that we were striving to create during the data collection procedures. Watching the video was not intended to be part of the therapy process or to developing therapy goals together, but only to select the MOIs. All therapists were used to the analysis of videos of their own therapeutic practice as a didactic and reflective tool during their training and were able to select three to five MOIs in each session. 

The large difference in duration between patient- and therapist-selected MOIs might seem surprising, but it is consistent with a long-standing discussion in music therapy about how to define a moment and how long a moment is. Further, what constitutes a moment is and how long it is, is not necessarily clear, as moments in therapy are related to participants and their therapists. Chronological measures do not capture what is discussed as kairologic experiences and the specific situated content of that process [24]. Therapists have come up with an array of potential descriptors, such as resonance, synchronicity, and affect attunement [28,61]), but the generalisation of what constitutes a moment has not yet been achieved and is based on different interests. However, a common denominator is that each of such moments, regardless of its duration, belongs to a therapeutic narrative of change [62], and there have been several attempts to systematise them. Wosch and Wigram [63] have discussed microanalytic approaches to define segments, episodes, and sections of the timeframes selected, and researchers have focussed on the musical content, for example, of particular motifs that engaged the patient and labelled them accordingly [64,65]. Here, we have accepted that patients offered us their specific choice of what they found interesting and also how long they attributed it to being interesting. The specific duration here is of interest for our time-based analyses of behavioural and neural processes.

### 4.2. Research Setting

Conducting research in a naturalistic setting obviously has trade-offs compared to a controlled setting in a laboratory, and requires a certain balance between what is possible and feasible in the field and what is interesting from a theoretical point of view. In our mixed method approach, we have tried to support phenomenological descriptions with quantitative data on a (neuro-)physiological level (EEG and ECG), to increase the validity of our qualitative data (participatory observation and interviews). Such an approach has already been realised in studies of healing settings “in the field” [23,66,67,68]. Qualitative research emphasises a natural setting and encourages data collection, including measurements, in those places where events take place in daily life [69,70]. 

In this project, we have tried to keep the music therapy and its setting in a rehabilitation centre as natural and authentic as possible. Nevertheless, there are obvious differences, since it is a therapy happening within the framework of a research project where data collection takes place at the same time, using various technical equipment and the presence of additional researchers. In the course of our participant observations, and according to the statements of the participants, wearing an EEG cap, amplifier, and ECG electrodes was only marginally distracting at the beginning of the first session of the therapy. However, the distraction gradually disappeared and had no remarkable impact on the process of the therapy itself. It was important for the participants, especially for the patients, to be properly informed about the research setting and what is going to happen, in order to get the feeling of “being in good hands”—this fact enabled them to completely engage in the therapy. It was noticeable and reported by the music therapist that essential therapy elements and themes took place, as is usually the case within a therapy session. For example, patients were able to open themselves up and talk about very personal topics, indicating that they experienced MT as a safe place. This observation encourages us that it is possible to study clinical reality with our approach.

However, bringing the ‘lab to the field” can also imply focussing on a common aspect of children’s music therapy in a laboratory research setting. A recent study [71] was creating an experimental setting that resembled a setting in which parents would join an MT session or watch a session behind an observation window while waiting. The researchers measured the dual-EEG of a child and the parent, while the parent watched the facial expressions of their child participating in the music therapy on a video screen in another room. The parents “had exclusively a frontal view of the faces of their children. This design was adapted to emulate current clinical practice” [71] (Samadani et al., 2021, p. 3). This research setting allowed good data recording control (at least on the side of the sitting parent) and enabled analysing the interbrain synchrony between parent–child dyads while parents were observing their child’s facial expressions. However, their study did not investigate therapist–patient interaction, but investigated an (non-interactive) aspect of the child–parent relationship in an experimental research setting situated in music therapy.

#### Rehabilitation Settings

Recruiting patients for this study was feasible. However, this feasibility study did not aim to assess a sufficient number of patients for a randomised controlled study. Rather, it aimed at assessing the feasibility of a lifeworld-orientated EEG–ECG hyperscanning procedure in clinical practice.

When scheduling music therapy research sessions, we made sure that patients did not miss other treatments. This was possible due to good cooperation with the persons responsible for therapy planning. However, data collection on three consecutive weeks was not always possible due to organisational constraints. For the main study, the holiday plans of the music therapists and scheduled medical examinations of the patients will be considered to ensure full data collection with each dyad. Two screened patients in the rehabilitation facility declined to participate due to the videography and participatory observation part of the research. However, enough patients fulfilled the inclusion criteria in the rehabilitation facility; therefore, the refusal of screened patients did not impede patient recruitment and the research schedule. In the rehabilitation ward of the university clinic, it took more time to find patients who fulfilled the inclusion criteria due to the severity of acute symptoms or patients being close to being discharged.

Regarding the practicability of this kind of research project, the rehabilitation facility, with its well-regulated daily routines, was more suitable than the acute hospital ward due to unpredictable medical events, examinations, and emergencies. In an acute medical setting, the focus is primarily on containing traumatic events and the assessment of medical, nursing, and acute-therapeutic issues, in which music therapy contributes. One session had to be interrupted due to a spontaneous medical examination of the patient, which was scheduled by the hospital during the time of data collection. Patients’ exact length of stay also could not be predicted, which impacted recruitment as well as data collection, and, as a result, the desired three consecutive weekly sessions.

An advantage for our project was that one of the researchers was familiar with both research settings. She had worked there before as a therapist, knew the team members and their responsibilities, as well as the organisational structure, and was able to consider potential challenges and pitfalls in the planning of the study. Staff from both organisations were involved in the planning phase to carefully and reasonably split tasks between members and to ensure the study ran smoothly. Before starting the feasibility study, the final processes of patient examination, assignment to music therapy, personal pre-consultation, and recruitment were presented to the teams, and we made sure that everybody knew what to do and how to find all the information needed for his/her task. In the rehabilitation clinic, continuous feedback during the course of the study was positive and the acceptance of the study was high.

### 4.3. EEG Technology

For the transportation of the equipment, two suitcases and several bags were used. Two members of the research team were present for each data collection. Compared to a lab setting, in which technological procedures can be controlled more easily, a mobile setting for each participant is prone to problems that may occur. However, these instances were not as often as expected. The technical realisation of this dual-EEG-ECG video setup for a real-life setting in a clinical ecology proved to be challenging. One promising synchronisation development with three small 4k cameras showed no reliable frame-based synchronisation results and could not be used for EEG-ECG video synchronisation. For our purposes, we were able to use the single video camera EEG setup provided by the EEG vendor. However, having multiple cameras (with different video perspectives on interactants) can be beneficial for detailed behaviour analyses that do not require highly accurate synchronisation.

EEG application, cap comfortability, and body-carried EEG amplifiers should not influence the therapy experience. However, one therapist experienced the setup as restricting, as she would have used more activities involving movement with her patient. The amplifiers were situated in the side-pockets of the Fisherman’s vests as shown in Figure 3, but it might be better to have vests, which would allow carrying the amps on the back in shoulder pockets; however, such vests were not available. During experience screening and interviews, none of the patients mentioned that the EEG procedures were exhausting for them. The transition from the gel caps to the saltwater caps saved time, as well as simplified the implementation of the research project for research staff and participants.

For the follow-up study, we plan to use the same analyses (evaluation of the before/after resting-state EEG, synchronisation of brain activity; for details, see the study protocol of the feasibility study in Fachner et al., 2021) [8]. As is explained elsewhere [23], we will focus on frontal alpha asymmetry as an indicator of emotional valence. 

Music therapy focuses on therapist–patient interactions, in which moments of empathy and emotional connection can occur [72], which then can promote motor rehabilitation and lead to an improvement in functional outcomes [73]. In a case study deriving from this feasibility cohort focussing on MOIs and their FAA timeseries [74], we were able to demonstrate how emotional processes, as shown in the FAA were aligning between therapist and patient, an observation that we described already earlier in a different therapy setting (Fachner, Maidhof et al., 2019) in which a strong emotional impact on the patient is shared with the therapist. The in-depth observation of this case study also confirms that we were able to record MOI EEG data in the field that is not only personally meaningful, but also has a sufficient data quality that allows further analyses. Apart from the analysis of shared emotional processing in the main study, we are interested in analysing directed brain-to-brain coupling [75], how MOIs and MONIs differ across sessions and dyads, and how this relates to the interactional content and emotional qualities.

### 4.4. Limitations and Outlook

Though receiving three weekly music therapy sessions reflects the rehabilitation pathways of our inpatients in the neurorehabilitation clinics, patients in other settings often receive more than three sessions, which in turn can alter patients’ experiences and the therapeutic relationship. It remains to be seen whether the acceptability of the research procedures might change over a longer course of music therapy. 

Although we are interested in dyadic processes, more than two persons were present in the music therapy room. This was methodologically necessary to allow for participatory observation (see Section 2.2.4 above), but none of the participants mentioned this as being distracting. Thus, we assume that the presence of other persons has a rather negligible influence on (music-therapeutic) dyadic processes.

Another more general limitation inherent in real-world approaches that investigate unconstrained, naturally occurring interactions, such as those in the present study, is the question of the functional significance and interpretability of EEG findings. Though these data have a high ecological validity, they presumably reflect a multitude of simultaneously occurring dyadic cognitive and affective processes that interact in complex ways. Nevertheless, our recent study [23] suggested that real-world (music therapy) data can be meaningfully interpreted, and future studies can combine this explorative and hypothesis-generating research with “naturalistic laboratory research” [76].

At these early stages of research, we can only speculate what the clinical applications could be. However, a potential optimisation of personalised music therapy assessment and delivery is conceivable, for example, with the help of automated detection of MOI instances based on neural descriptors of patient engagement, therapeutic insight, emotional intensity, and regulation. We agree that “an understanding of neural synchronization processes during music therapy sessions could be a crucial and viable tool for high-quality therapy sessions” (Kang et al., 2022, 5f) [77]. The clinical implication of process- and outcome-related social neuroscience research for the horizon of personalised medicine and music therapy seems thus to be manifold and promising, in various clinical fields. This is in line with a recent position paper, claiming that the use of EEG hyperscanning in individuals with neurological diagnoses, such as stroke, is “particularly relevant and could provide additional insight on neural dynamics, optimising rehabilitation strategies for each individual patient” (Short et al., 2021, p. 1) [78]. Whereas these authors focus on the advantage of investigating functional gains of interventions with hyperscanning, we are demonstrating how EEG hyperscanning can be used for process research in a naturalistic music therapy setting delivering active music therapy that focuses on the actual personal needs of the patient.

## 5. Conclusions

In this study, we reported on the feasibility of implementing a mobile EEG hyperscanning and AV lab into a naturalistic clinical setting of music therapy in neurorehabilitation. To bring the ‘lab into the field’, we had to adapt some technical procedures, and we tested different AV data recordings to arrive at a feasible level of data quality. The recruitment of participants and implementing the research setting were easier in a neurorehabilitation clinic, because there are different routines and demands compared to an acute setting. The acceptability of the research in the rehabilitation clinic was high, and the staff was open to further studies. Patients did not report any extra burden placed on them, experienced the research as a welcomed diversion from their daily hospital routine, felt safe and secure with the team, and were happy to be part of a research project that may help other patients in the future. Thus, future research with this approach in rehabilitation settings seems feasible, and further research in this domain is warranted.

## Figures and Tables

**Table 1:** Planned and actual duration of study procedures.

Study Procedures	Description	Planned Duration (Approx.)	Recorded Average Duration (Patients)
Preparation	Introduction, EEG + ECG preparation, therapy readiness questionnaire	60 min	47 min (21 min, 90 min)
Music therapy with EEG measurements, ECG measurements + video recording	Therapy session as in normal practice (max. 30 min.) 5 min. resting state EEG before and after session	40 min	37 min (26 min, 50 min)
EEG measurements	Short synchronised playing sequence (2 × 3 Min.) including drum setup following resting state EEG	6 min (2 × 3 min)	8 min (7 min, 11 min)
Experience screening	Open questions about experiences and perceptions of the therapy session	15 min	5 min (4 min, 8 min)
BREAK	Washing hair, eating, recovery	90 min	86 min (73 min, 101 min)
Video ratings	Choosing 3 MOIs and at least 1 MONI from the video	45 min	41 min (36 min, 55 min)
MOI Interview + identify moment of meeting + identify MONI Feasibility interview	Qualitative interview about chosen MOIs; patient asked to identify moments of meeting and MONIs	30 min	18 min (5 min, 33 min) (MOI interview only)
	Total duration (excluding break):	196 min	155 min (94 min, 246 min)

MOI—Moment of Interest, MONI—Moment of No Interest.

**Table 2:** Patient/therapist characteristics, technology used, retention and responses.

Patient Sex/ Age/ Handedness	Post-Stroke Time	Stroke Areas	Recruitment Site	Numbers of Sessions (S)	EEG Cap	Reason for Sessions Missing	Therapist Sex/ Age/ Handedness	Feasibility Q1 Experience of Research Setting	Feasibility Q2 Questions and Instructions	Feasibility Q3 Use of Computer and MOI Selection	Feasibility Q4 Participate in Future/Recommend to Others?	Synchronous Drumming
m, 64 y, right-handed	29 months	Media infarct (ICD-10 I69.3)	rehab	3	Gel (Saline 3rd Session)	N/A	MT 1, w, 41 y, right-handed	professional, duration is ok, am in good hands	well explained, clear (comprehensible)	PC: Difficult at first but well instructed MOI selection: ok, lengthy	yes, thankful for opportunity/ yes	Not in Session 1
m, 48 y, right-handed	7 months	Posterior and thalamus infarct (ICD-10 I63.9)	rehab	2	Gel	organisational reasons annual leave of therapist	MT 1, w, 41 y, right-handed	Comfortable, no stress	clear, comprehensible	PC: normal MOI selection: ok	yes, interesting/ yes	Not in Session 1
m, 79 y (mixed handed)	2 weeks	right parietal, mild left parietal and right frontal	acute	1	Saline	intermission for acute investigation (1st session cancelled); transferral	MT 2, w, 25 y, right- handed	Interesting and varied	clear, comprehensible	Interesting, technically interesting	N/A	Not in Session 2
m, 58 y, right-handed	9 months	Ischemic stroke, partial MCA infarction, A. pericallosa infarction (ICD-10 I69.3)	rehab	2	Saline	Planned holiday; discharge	MT 3, w, 24 y, mixed-handed	Very good, exciting the first time round	clear, comprehensible	manageable	Yes, without hesitation	Not in Session 1

ICD—International classification of diseases, MCA—middle cerebral artery, MT—music therapist, PC—personal computer, MOI—moment of interest.

**Table 3:** Patients’ MOI/MONI selections and durations.

Patient/Session	Number of Selected MOIs	MOIs: Average Duration (Min-Max) [s]	Number of MOI Overlaps (Patient-Therapist)	Average Duration of Overlaps (Min-Max) [s]	MONI: Duration Each [s]
FD1/S1	5	5 (1–13)	2	1 (1)	3
FD1/S2	9	8 (1–22)	3	3 (1–7)	4
FD1/S3	10	24 (2–130)	-	-	-
FD2/S1	4	8 (2–16)	1	16	-
FD2/S2	4	15 (10–31)	2	20 (9–31)	-
FD3/S2	5	82 (10–140)	1	0.5	-
FD4/S1	4	76 (35–158)	3	19 (3–35)	30; 147
FD4/S2	7	85 (57–120)	2	14 (12–16)	120

MOI—Moment of interest, MONI—moment of no interest.

## Data Availability

Not applicable.

## Appendix A. Log Sheet–MOI Feasibility

TN-Code Patient: _________________ Therapist:____________________

□ University clinic □ Neurorehabilitation clinic

Date: _________________________

Timings

	Start	Finish
Total duration		
Equipment set-up		
Greetings and patient information		
EEG/ECG application		
Music therapy session		
Experience screening		
Video rating		
MOI Interview		

## Appendix B. Feasibility Questions for Participants

(1) How did you just experience the research setting?
(2) Were our instructions and questions comprehensible for you?
(3) Was working with the computer while watching the videos and selecting the MOIs acceptable for you?
(4a) Would you take in part in another study like this in the future, and if not, why?
(4b) Would you encourage others to participate? If not, why?

Any arising difficulties or observations:

Reflection and notes:

## Appendix C. Interview Guide

Experience screening after the session:

- How did you just experience the therapy situation? How did it go for you? Tell us freely about what comes to mind.
- Additional question: Is there anything else you would like to say about this?
- Closing question: How did you experience the research setting (the environment, instructions, etc.…)?

Instructions for the video rating

At least three, up to five maximum moments of interest should be selected.

- Choose at least three (at most five) moments from the videos that were interesting for you. You will subsequently be asked about three of these moments (why you precisely chose these moments and what was interesting about them).

Interviews about the three MOIs selected during the video rating:

- Please describe the three selected “interesting moments”; what was interesting about these and why did you precisely choose these moments?
- Additional question: Were there any moments from the ones you chose that you would describe as a “moment of meeting”? (i.e., where you had the feeling there was a special connection between you and the other person).
- If not, could you describe a (different) moment of meeting for me?

Interview about the MONI during the video rating:

- Now please look for one more moment, that does not seem as interesting to you–in contrast to the interesting moments.
- Please describe why this moment does not seem interesting.
